# Cost of hospital care of women with postpartum haemorrhage in India, Kenya, Nigeria and Uganda: a financial case for improved prevention

**DOI:** 10.1186/s12978-020-01063-x

**Published:** 2021-01-22

**Authors:** Fiona Theunissen, Isotta Cleps, Shivaprasad Goudar, Zahida Qureshi, Olorunfemi Oludele Owa, Kidza Mugerwa, Gilda Piaggio, A. Metin Gülmezoglu, Miriam Nakalembe, Josaphat Byamugisha, Alfred Osoti, Sura Mandeep, Teko Poriot, George Gwako, Sunil Vernekar, Mariana Widmer

**Affiliations:** 1 Concept Foundation, Avenue de Sécheron 15, Geneva, Switzerland; 2 KLE Academy of Higher Education and Research, J N Medical College, Belagavi, Karnataka India; 3 Department of Obstetrics and Gynaecology, School of Medicine, University of Nairobi, Nairobi, Kenya; 4 Department of Obstetrics & Gynaecology, Mother & Child Hospital, Akure, Nigeria; 5 Department of Obstetrics and Gynecology, School of Medicine, Makerere University College of Health Sciences, Kampala, Uganda; 6 Statistika Consultoria, Campinas, Brazil; 7 Department of Reproductive Health and Research, World Health Organization, UNDP/UNFPA/UNICEF/WHO/World Bank Special Programme of Research, Development and Research Training in Human Reproduction (HRP), Avenue Appia 20, 1201 Geneva, Switzerland

**Keywords:** Postpartum haemorrhage, Oxytocin, Heat stable carbetocin, PPH, Cost

## Abstract

**Objective:**

Access to quality, effective lifesaving uterotonics in low and middle-income countries (LMICs) remains a major barrier to reducing maternal deaths from postpartum haemorrhage (PPH). Our objective was to assess the costs of care for women who receive different preventative uterotonics, and with PPH and no-PPH so that the differences, if significant, can inform better resource allocation for maternal health care.

**Methods:**

The costs of direct hospital care of women who received oxytocin or heat-stable carbetocin for prevention of PPH in selected tertiary care facilities in India, Kenya, Nigeria, and Uganda were assessed. We collected data from all women who had PPH, as well as a random sample of women without PPH. Cost data was collected for the cost of stay, PPH interventions, transfusions and medications for 2966 women. We analyzed the difference in cost of care at a facility level between women who experienced a PPH event and those who did not.

Key findings

The mean cost of care of a woman experiencing PPH in the study sites in India, Kenya, Nigeria, and Uganda exceeded the cost of care of a woman who did not experience PPH by between 21% and 309%. There was a large variation in cost across hospitals within a country and across countries.

**Conclusion:**

Our results quantify the increased cost of PPH of up to 4.1 times that for a birth without PPH. PPH cost information can help countries to evaluate options across different conditions and in the formulation of appropriate guidelines for intrapartum care, including rational selection of quality-assured, effective medicines. This information can be applied to national assessment and adaptation of international recommendations such as the World Health Organization’s recommendations on uterotonics for the prevention of PPH or other interventions used to treat PPH.

*Trial registration* HRP Trial A65870; UTN U1111-1162-8519; ACTRN12614000870651; CTRI/2016/05/006969, EUDRACT 2014–004445-26. Date of registration 14 August 2014

**Plain English summary:**

Access to quality, effective lifesaving medicines in low and middle-income countries remains a major barrier to reducing maternal deaths from bleeding after childbirth. Information on to what extent treatments for bleeding increases the cost of care of women after childbirth is important for informed resource allocation.

We collected data from all women who had bleeding after childbirth, as well as a random sample of women without bleeding in selected hospitals in India, Kenya, Nigeria, and Uganda. Cost data was collected for the cost of stay and interventions to manage bleeding for 2966 women. We compared the difference in cost of care between women who experienced a bleeding event and those who did not.

The mean cost of care of a woman with bleeding in the study sites exceeded the cost of care of a woman who did not experience PPH by between 21% and 309%. There was a large variation in cost across hospitals within a country and across countries.

Our results indicate an increased cost of bleeding of up to 4.1 times that for birth without bleeding. Effective prevention reduces the cost of care. Cost information can help countries to evaluate options across different conditions and in the formulation of appropriate guidelines for intrapartum care, including rational selection of quality-assured, effective medicines. This information can be applied to national assessment and adaptation of international recommendations such as the World Health Organization’s recommendations on medications for the prevention of bleeding after childbirth or other interventions used to treat bleeding.

## Background

Postpartum haemorrhage (PPH) is a leading cause of maternal mortality, globally, disproportionately affecting low and middle-income countries (LMIC) [1, 2]. It is also a significant contributor to severe maternal morbidity and long-term disability [3–5]. Preventing PPH is essential for improving maternal health outcomes on the path to the achievement of the United Nations Sustainable Development Goals and ensuring sound financial stewardship to support Universal Health Care efforts [6].

PPH caused by uterine atony can be prevented to a large extent through the administration of a quality-assured, effective uterotonic immediately following the birth of the baby [2].

The World Health Organization’s (WHO) first line recommendation for the prevention of PPH is the administration of oxytocin 10 IU [7], an injectable uterotonic which requires refrigeration during transportation and storage to remain effective [8]. Heat-stable carbetocin has clinical and pharmacological properties similar to those of oxytocin but does not require refrigeration, retaining its efficacy even after exposure to high temperatures for long periods of time [9]. WHO recommends heat-stable carbetocin for PPH prevention in settings where oxytocin is unavailable or the quality might be compromised due to heat degradation, and where its cost is comparable to other effective uterotonics [7 p. ix].

Currently, decision makers only have access to information on uterotonic prices. They lack information on the total cost of facility-based care of women with and without PPH. We conducted an ancillary study using data from WHO CHAMPION trial (Heat-stable carbetocin versus oxytocin to prevent hemorrhage after vaginal birth) sites [10], to assess the direct hospital care costs of women in nine tertiary referral hospitals in India, Kenya, Nigeria, and Uganda, to provide evidence to support more informed decision making on options for PPH prevention in LMIC. These countries contribute significantly to the global maternal morbidity and mortality burden. Our focus in this ancillary study is restricted to PPH prevention options in facility-based births and does not cover births outside health facilities.

## Methods

### Study aims, design and population

We calculated the cost of hospital care of women with and without PPH and who received either oxytocin 10 IU or heat-stable carbetocin 100 µg as part of the management of the third stage of labour at nine tertiary referral hospitals (sites) in India, Kenya, Nigeria and Uganda.

The CHAMPION trial was a randomized, double-blind, non-inferiority trial comparing the effectiveness in the prevention of PPH of an intramuscular injection of heat-stable carbetocin with oxytocin administered immediately after vaginal birth. The trial methods and results are described in detail elsewhere [10]. Briefly, almost 30,000 women across 23 sites in ten countries were randomly assigned to prophylactic heat-stable carbetocin or oxytocin. The primary outcomes were the proportion of women with blood loss of at least 500 ml or the use of additional uterotonic agents, and the proportion of women with blood loss of at least 1000 ml. Secondary outcomes included the proportion of women having additional interventions to control bleeding. The trial results showed that heat-stable carbetocin was non-inferior to oxytocin.

**Fig. 1:**
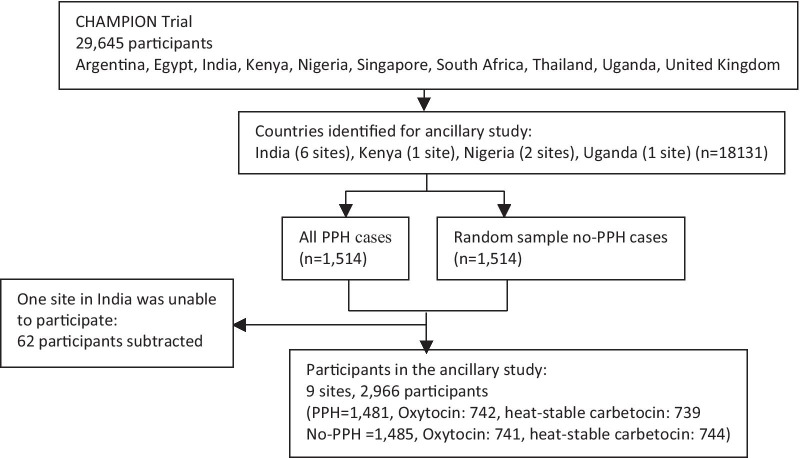
Study flow chart

Data from all women who participated in the CHAMPION trial in India, Kenya, Nigeria, and Uganda and who had PPH (n = 1514), as well as a random sample of the same number of women who did not experience PPH (n = 1514), was extracted from the CHAMPION trial database (Fig. 1). One of the ten trial sites was eliminated from this study due to administrative barriers to data collection (n = 62). A total of 2966 records from the CHAMPION trial were therefore included in this study. The four countries were selected because of their high maternal mortality and morbidity rates, climate, and possible difficulties of ensuring the quality of oxytocin due to challenges maintaining the cold chain of oxytocin.

### Cost calculations

The following data, covering the time immediately following administration of the prophylactic uterotonic to the time of discharge from the hospital, were extracted from the CHAMPION trial database to form the basis of the calculation of the cost of care: administration of additional uterotonics; other medical and surgical interventions to treat PPH; blood transfusion and duration of hospital stay. The medical and surgical interventions recorded in the CHAMPION trial patient records and costed in this study were: suturing of the cervix/high vaginal tear; intrauterine balloon/condom tamponade; exploration of the uterine cavity; uterine or hypogastric ligation; manual removal of the placenta; bimanual compression of the uterus and hysterectomy. Uterine compression sutures and manual or surgical correction of uterine inversion were not used for any cases at any of the sites and therefore are not reflected in the results.

Excel data collection worksheets were specifically designed for this exercise, modelled on those developed by the Guttmacher Institute for their study estimating the cost of PPH in Egypt [11]. The worksheet design was reviewed by an obstetrician with experience in low income countries and by a local expert panel established in each country by the coordinator of the CHAMPION trial. The correspondent costs were obtained by the CHAMPION trial investigators from each of the participating hospitals. Sites obtained data from various sources including hospital pharmacies, accounting departments, payroll, and government salary data. Table 1 lists all elements included in each of the items costed for the study.

**Table 1:** Breakdown of costs and details of calculations

Description	Calculation
Medical and surgical interventions (I)	Cost of disposable apparel (gowns, gloves, caps etc.) + disposable consumables (needles, gauze, syringes etc.) + instruments (sterilization fee) + cleaning + laboratory tests + fee for use of operating theatre (if applicable) + staff labour cost (L) Each surgical intervention was costed as an independent intervention, although some patients may have received more than one intervention concurrently with some costs shared across interventions
Staff labour during interventions (L)	Monthly salary/hours worked per month x hours devoted to intervention Calculated for all personnel required for the procedure
Drug tray (DT)	Total price of the full tray of drugs available for the surgical intervention
Blood transfusion (B)	First unit of blood*:* Cost of apparel + consumables + sterilization charge + laboratory tests + blood product price + cost of labour for blood (LB) Each additional unit of blood*:* Cost of labour for blood (LB) + blood product price
Hospital stay (HS)	Basic cost of 24 h stay in ward bed x the number of days in hospital from the time of birth to discharge Some patients were discharged within 24 h and with no additional care over basic delivery services. As we did not calculate the cost of delivery specifically (it occurs prior to diagnosis of PPH) these patients’ records would show zero cost. To avoid this, all patients were allocated a minimum of 1 day’s hospital stay as a proxy for the cost of delivery
Additional uterotonics (AU)	Price of the dose of the uterotonic administered, being either: the price of the individual unit (i.e. tablet, ampoule) multiplied by the units administered or the pack price divided by units in the pack multiplied by the units administered
Concomitant medicines (CM)	Price of the dose of the medicine administered, being either: the price of the individual unit (i.e. tablet, ampoule) multiplied by the units administered or the pack price divided by units in the pack multiplied by the units administered
Concomitant formula *T*2 = *HS* + Σ*I* + *U* + *B* + *CM*	T2 = Total cost of care HS = Cost of hospital stay I = Costs of a surgical intervention received for treatment of PPH and ΣI = the sum of costs of all surgical interventions received U = Sum of the cost of all additional uterotonics administered for treatment of PPH B = Cost of blood and blood products administered CM = Sum of cost of all concomitant medicines administered
Drug tray formula *T*3 = *HS* + Σ(*I* + *DT*) + *U* + *B*	HS = Cost of hospital stay I = Costs of a surgical intervention received for treatment of PPH and DT = Cost of the full tray of drugs available for each surgical intervention for PPH treatment U = Sum of the cost of all additional uterotonics administered for treatment of PPH B = Cost of blood and blood products administered

The data for concomitant medicines administered during the CHAMPION trial included an extensive list of medicines, many of which were not related to PPH care. It was not possible to isolate the drugs that were administered specifically for the treatment of PPH from the list of concomitant drugs. The cost of the drugs available for each surgical intervention was also collected to complement the cost information. Investigators from each of the countries were requested to list all the drugs used to treat PPH. This list developed per country was named ‘drug tray’ for the purposes of this study

The Drug Tray formula was used to validate the results by addressing the uncertainty about the concomitants being related to PPH or not. For the Drug Tray formula, it should be noted that the entirety of the drug tray may not have been used for each intervention depending on each patient’s medical condition. It was not logistically possible to cross-match the concomitants and drug tray data to achieve a more accurate result

All prices collected in this study were current at the time of data collection, i.e., between February and July 2018, and not at the time of the CHAMPION trial intervention. The conversion from local currency to US Dollar was made at the time of data collection. Disposable consumables were costed using unit prices provided by the facilities from procurement records. The costs related to the use of non-disposable items, such as surgical instruments, were calculated as the cost of cleaning and sterilization. We did not include depreciation as information on the life of non-disposable items was not available.

One on-site meeting with each country investigator was performed by the research team during the data collection period for quality assurance purposes.

The total cost of care at the facility for each patient (PPH and no PPH) was computed using three different formulas. These were (1) Total cost of care; (2) Total cost of care including concomitant medicines women received; and (3) Total cost of care including drug tray costs. We did not include the concomitant medicines and drug tray formulas in the main manuscript since, due to variations in the number of medicines used and the contents of the drug trays, the results showed high variability and distracted from the main comparison objective. The concomitant medicines and drug tray formulas are presented in Table 1.

The formula used for the total cost of care calculation was:

$$T1 = HS + \Sigma {\text{I}} + U + B$$

where T1 = Total cost of care, HS = Cost of hospital stay, I = Costs of a surgical intervention received for treatment of PPH and ΣI = the sum of costs of all surgical interventions received, U = Sum of the cost of all additional uterotonics administered for treatment of PPH, B = Cost of blood and blood products administered.

The cost of prophylactic uterotonics (oxytocin or heat-stable carbetocin) was not included as their administration occurred prior to diagnosis of PPH.

The mean costs of care for women receiving oxytocin or heat-stable carbetocin during the third stage of labour, having PPH or not, were calculated for each site but are not presented in the paper since the two uterotonics were very similar in efficacy.

## Results

The hospitals that participated in this sub-study of the CHAMPION trial were all tertiary level referral facilities with full time obstetrician and gynaecologist and midwife presence. The two hospitals in Nairobi, Kenya and Kampala, Uganda are large tertiary facilities with approximately 10,000 and 33,000 annual births, respectively. The Nigerian hospitals had approximately 2500 births and the Indian facilities had between 1200 to 3500 births annually. All facilities have emergency surgery and blood transfusion services.

Women with PPH were more likely to have risk factors for PPH such as increased labour augmentation, instrumental birth, previous caesarean section and PPH (Table 2).

**Table 2:** Baseline variables by PPH status

Baseline variable	No PPH N = 1485		PPH N = 1481		All N = 2966	
	n	(%)	n	(%)	n	(%)
Nulliparous	735	49.5	785	53.0	1520	51.2
Labor induced	179	12.1	188	12.7	367	12.4
Labor augmented	642	43.2	761	51.4	1403	47.3
Instrument-assisted vaginal birth	34	2.3	79	5.3	113	3.8
Perineal trauma leading to suture	1011	68.1	1074	72.5	2085	70.3
Baby alive	1452	97.8	1460	98.6	2912	98.2

	Mean	SD	Mean	SD	Mean	SD
Mother age (years)	25.3	4.8	25.8	5.2	25.5	5.0
Gestational age (completed weeks)	38.4	2.3	38.7	2.0	38.6	2.1
Birth weight (g)	2881	548	3133	551	3007	563

Baseline variable among parous women	No PPH N = 750		PPH N = 696		All parous women N = 1446	
	n	(%)	n	(%)	n	(%)
CS in previous pregnancies	39	5.2	56	8.0	95	6.6
PPH in previous pregnancies	6	0.8	39	5.6	45	3.1

### Cost of care for women with PPH and no-PPH

In all sites, PPH represents an increase in the cost of care for women. This increase ranged from 21% in the Uganda site to 309% at one of the Nigerian sites. The increase in the cost of PPH using the concomitants and drug tray formula followed similar trends although the variations across hospitals were larger (Table 3).

**Table 3:** Increased cost of care of PPH over No PPH per woman in USD

Hospital	Mean cost of care (USD)		Cost ratio of PPH vs no PPH (95% CI)
	No PPH	PPH	
India-BLDE Hospital	9.28	11.04	1.2 (1.0 to 1.4)
India-Dr. Sujata College	11.14	13.30	1.2 (1.0 to 1.4)
India-KLES Hospital	5.76	12.11	2.1 (1.7 to 2.5)
India-Lata Med. Research Hospital	12.30	15.46	1.3 (0.9 to 1.7)
India-NM College	5.25	6.96	1.3 (1.1 to 1.6)
Kenya	22.12	34.36	1.6 (1.4 to 1.7)
Nigeria-Mother & Child Hospital Akure	2.39	6.60	2.8 (2.4 to 3.2)
Nigeria-University of Ibadan	17.42	28.84	1.7 (1.4 to 2.0)
Uganda	13.37	14.85	1.1 (1.0 to 1.3)

As the sites were not randomly selected as representative of all facilities in the four study countries, we have not calculated an average increased cost of care of PPH over no PPH for each country. Detailed findings for each intervention and uterotonics prices are included in Additional file 1: Annexes.

The most common interventions were suturing of the cervix/high vaginal tear and blood transfusions. The most frequently used additional uterotonics were oxytocin and misoprostol. In Table 4, we present the drivers of the costs. Hospital stay contributes most to the cost. The figures show a skewed distribution with few very high values in those patients with PPH. It is likely that some of the severe PPH patients receive multiple interventions and stay in hospital longer.

**Table 4:** Consumption of health resources by PPH in USD

	No PPH (n = 1485)						PPH (n = 1481)					
	Min	Q1	Median	Mean	Q3	Max	Min	Q1	Median	Mean	Q3	Max
Cost stay in hospital	1.4	6.6	11.8	12.5	16.2	61.2	1.2	4.7	10.9	12.9	16.2	106.3
Cost uterotonics	0.0	0.0	0.0	0.1	0.0	6.1	0.0	0.0	0.0	1.2	2.8	87.5
Cost interventions	0.0	0.0	0.0	0.5	0.0	191.9	0.0	0.0	0.0	10.4	0.0	857.6
Cost transfusions	0.0	0.0	0.0	0.3	0.0	49.3	0.0	0.0	0.0	2.4	0.0	198.9
Total cost without concomitants	1.4	6.6	12.2	13.4	16.2	219.4	1.2	7.3	15.6	26.9	24.4	1145.0

Four of the sites studied showed increases exceeding 100% in cost of care for PPH cases over no PPH with all three formulas. The clinical management of cases varied across settings. The Belgaum site saw the highest frequency of blood transfusion (28.07%) and the second highest frequency of suturing of the cervix/high vaginal tear (12.28%), bimanual compression (5.26%) and exploration of the uterine cavity (3.51%). The particularly high level of blood transfusion at this site, which may be related to the high levels of pre-existing anaemia found in pregnant women in India, was a major contributor to the increase in cost of care. The site also had high levels of uterotonic use, including high use of carboprost which was the most expensive uterotonic. (Carboprost was only used in India). The Nairobi, Kenya site had a moderate rate of surgical intervention, but the highest use of additional uterotonics. Blood transfusions were particularly costly at this site. The Akure Nigeria site had the highest level of oxytocin and misoprostol use with comparatively high prices for both uterotonics. The site also had the highest number of surgical interventions, relatively high frequency of suturing of the cervix/high vaginal tear (11.46%) and blood transfusion (11.86%) and the highest use of uterine balloon/condom tamponade (7.51%). The Ibadan, Nigeria site had the highest frequency of suturing of the cervix/high vaginal tear (16.8%) and moderately high use of oxytocin and misoprostol with high prices for both uterotonics.

## Discussion

### Main findings

Our results quantify the increased cost of managing women with PPH of up to 4.1 times that of a birth without PPH although there was variability across settings. This increased financial burden is a result of the fact that the management and treatment of PPH requires a range of medicine, surgical, and other interventions, all of which require human and other resources, including an effective supply chain. There was a large variation in cost across hospitals. PPH management for reasons other uterine atony requires additional costs such as the use of sutures for tears or antibiotics for retained placenta. However, uterotonics remain the mainstay of PPH management. We note that PPH cases are associated with increased interventions during labour such as augmentation, instrumental delivery and episiotomy and tears. It is likely therefore that over-medicalization is also associated with increased costs.

### Strengths and limitations

This ancillary study was conducted at nine sites in four countries with high maternal mortality rates and calculated the cost of care of 2966 participants in total, making it the largest PPH cost of care assessment at the time of study.

We used data recorded from the CHAMPION trial, whose aim was to compare the clinical efficacy of two uterotonics (oxytocin and heat-stable carbetocin) for the prevention of PPH, but not to determine the cost of care. The design of this ancillary study was therefore limited by the data categories of the primary study. Notwithstanding this, the CHAMPION study data set is extensive, covering all interventions received as a result of a diagnosis of PPH, providing a unique opportunity to calculate the cost of care for a large cohort of patients.

The facilities in the study are all referral hospitals in urban settings, although they vary in size and capacity and the settings themselves vary considerably. The data may not be fully representative of the cost of care of women delivering in other levels of healthcare, such as maternity clinics, primary or secondary level hospitals. It is not possible to determine from this study whether the cost of care for a woman delivering in a rural or secondary level facility would be higher or lower than the costs we have calculated in this study. While some costs may be reduced, such as the cost of labour, additional costs are likely to be incurred in the event of PPH such as the cost of obtaining blood from a referral facility, the cost of patient transfer to a higher level of care and the cost of additional care to stabilise a patient for transfer. A patient experiencing PPH at a lower level of healthcare, where access to expert care and surgical intervention is limited, may also result in additional interventions when they arrive at the referral facility, due to the time lost in transfer. All patients in this study were delivered at the study site. There were no after-birth referrals from other facilities.

Converting the cost data obtained into United States Dollars (USD—using the exchange rate current at the time of data collection) enabled us to calculate an overall additional cost for PPH over no PPH care. While this conversion does not consider different health system characteristics, the increased cost would still give an idea about the likely differences in scale.

We demonstrated that in facilities across the four countries included in the study, the cost of care of a woman who experienced PPH was higher than the cost of care of a woman with no PPH, the ratio varying from 1.2 times higher (in Uganda) to 4.1 times higher (in a hospital in Nigeria), so that the ratio of costs depended on the setting. The variations across facilities could in part be due to differences in the interventions used to treat PPH. We collected extensive data on the concomitant medicines and the content of drug trays for women requiring surgical procedures. Unfortunately, the substantive amount of concomitant medicines had nothing to do with PPH related care and it was not possible to separate the medicines on a case-by-case basis. Similarly, the drug tray contents also varied, and those two formulas were not very helpful in the interpretation of the results. The high heterogeneity in costs also suggests that the facilities were not following similar guidelines and that could be emphasized to improve the quality of care.

Only atonic PPH can be prevented through the administration of a prophylactic uterotonic. This study included all cases of PPH from the CHAMPION trial (in the four countries), not just those caused by atony (failure of the uterus to contract). As the cause of PPH was not recorded for all participants, it was not possible to isolate atonic PPH cases to accurately determine the savings that could be achieved through more effective prevention of PPH, although the data could be used for such analysis.

The CHAMPION trial results showed that the risk of PPH was practically the same when both drugs are manufactured, distributed, and stored under optimal conditions [10]. The authors noted that the stability of heat stable carbetocin is likely to provide benefits in settings where a cold chain cannot be assured from manufacturer to patient. Although initially we intended to compare the costs between oxytocin and heat stable carbetocin, we did not include this analysis in the main body of the paper because the clinical effects were similar with both (this information is available in Additional file 1: Annex).

Countries need information on the prices of medicines, distribution and storage costs and costs related to the care of patients to make informed decisions regarding purchasing across different areas of health care. All uterotonic options available in these countries were available at low prices (see Additional file 1: Annex), relative to the cost of care of the PPH patients. Where the purchase prices of the uterotonic options are similar, the cost of distribution and storage plays a greater role in the overall financial impact. There is extensive evidence of widespread poor quality of oxytocin due to low-quality manufacturing and cold-chain deficiencies across LMICs with resulting negative impacts on the outcomes for prevention of PPH [12, 13]. Because of the high cost of care of patients experiencing PPH, countries could reduce the overall financial burden associated with childbirth care by investing in the uterotonic most likely to be effective at the time of use, as well as other evidenced-based practices.

## Conclusion

There is an important increase in the cost of care of women with PPH when compared to women without PPH.

The countries involved in this study could use the cost information, along with other relevant information such as morbidity impact data to evaluate all options for intrapartum care. Other countries should consider collecting and using similar data to better inform policy and practice decisions.

## Supplementary Information

**Additional file 1.** Annex: Detailed cost data.

## Data Availability

The data and analyses are available from the author for correspondence on request. The original trial data are available through the WHO web site.

## Abbreviations

CHAMPION trial: Carbetocin haemorrhage prevention trial
LMIC: Low and middle-income countries
PPH: Postpartum haemorrhage
USD: United States Dollar
WHO: World Health Organization
