# Correction to: Cost of hospital care of women with postpartum haemorrhage in India, Kenya, Nigeria and Uganda: a financial case for improved prevention

**DOI:** 10.1186/s12978-021-01110-1

**Published:** 2021-03-06

**Authors:** Fiona Theunissen, Isotta Cleps, Shivaprasad Goudar, Zahida Qureshi, Olorunfemi Oludele Owa, Kidza Mugerwa, Gilda Piaggio, A. Metin Gülmezoglu, Miriam Nakalembe, Josaphat Byamugisha, Alfred Osoti, Sura Mandeep, Teko Poriot, George Gwako, Sunil Vernekar, Mariana Widmer

**Affiliations:** 1 Concept Foundation, Avenue de Sécheron 15, Geneva, Switzerland; 2 KLE Academy of Higher Education and Research, J N Medical College, Belagavi, Karnataka India; 3 Department of Obstetrics and Gynaecology, School of Medicine, University of Nairobi, Nairobi, Kenya; 4 Department of Obstetrics & Gynaecology, Mother & Child Hospital, Akure, Nigeria; 5 Department of Obstetrics and Gynecology, School of Medicine, Makerere University College of Health Sciences, Kampala, Uganda; 6 Statistika Consultoria, Campinas, Brazil; 7 Department of Reproductive Health and Research, Development and Research Training, World Health Organization, UNDP/UNFPAUNICEF/WHO/World Bank Special Programme of ResearchHuman Reproduction (HRP), Avenue Appia 20, 1201 Geneva, Switzerland

## Correction to: Reprod Health (2021) 18:18 10.1186/s12978-020-01063-x

Following publication of the original article [1], the authors identified some errors in the text. The corrected text parts are mentioned below:

## Abstract

The mean cost of care of a woman experiencing PPH in the study sites in India, Kenya, Nigeria, and Uganda exceeded the cost of care of a woman who did not experience PPH by between 10 and 180%.

Our results indicate an increased cost of bleeding of up to 2.8 times that for birth without bleeding.

## Results

This increase ranged from 10% in the Uganda site to 180% at one of the Nigerian sites (Page 5 of 8, column 1).

Two of the sites studied showed increases exceeding 100% in cost of care for PPH cases over no PPH with all three formulas (Page 6 of 8, column 1).

## Discussion

Our results quantify the increased cost of managing women with PPH of up to 2.8 times that of a birth without PPH although there was variability across settings (Page 6 of 8, column 1).

We demonstrated that in facilities across the four countries included in the study, the cost of care of a woman who experienced PPH was higher than the cost of care of a woman with no PPH, the ratio varying from 1.1 times higher (in Uganda) to 2.8 times higher (in a hospital in Nigeria), so that the ratio of costs depended on the setting (page 7 of 8).
