# Supplementary material for: Cost of hospital care of women with postpartum haemorrhage in India, Kenya, Nigeria and Uganda: a financial case for improved prevention
Source: Reprod Health. 2021 Jan 22;18:18. doi: 10.1186/s12978-020-01063-x (PMC7821537; doi:10.1186/s12978-020-01063-x)
Supplement: Supplementary file 1 — Additional file 1. Annex: Detailed cost data. [file 12978_2020_1063_MOESM1_ESM.docx]

## Annexes

#### ANNEX 1 - COST DATA PER SITE

Table S1a: Increased cost of care of PPH over No PPH per woman using concomitants formula in local currency

| **Country** | **Hospital** | **Local Currency** | **Mean Cost of Care - No-PPH** | **Mean Cost of Care – PPH** | **Increased cost of care for PPH over No-PPH** | **% Increase in Cost of Care** |
| --- | --- | --- | --- | --- | --- | --- |
| India | Bagalkot | INR | 908·62 | 1,373·17 | 464·55 | 51% |
| India | Belgaum | INR | 895·06 | 2,236·30 | 1,341·24 | 150% |
| India | Bijapur | INR | 1,419·39 | 1,896·67 | 477·28 | 34% |
| India | Cuttack | INR | 1,743·24 | 1,493·84 | - 249·40 | -14% |
| India | Nagpur | INR | 934·91 | 1,383·74 | 448·84 | 48% |
| Kenya | Nairobi | KES | 2,521·88 | 6,797·57 | 4,275·69 | 170% |
| Nigeria | Akure | NGN | 1,850·28 | 6,155·73 | 4,305·45 | 233% |
| Nigeria | Ibadan | NGN | 9,917·36 | 21,345·56 | 11,428·20 | 115% |
| Uganda | Kampala | UGX | 53,846·15 | 65,769·23 | 11,923·08 | 22% |

Table S1b: Increased cost of care of PPH over No PPH per woman using the drug tray formula in local currency

| **Country** | **Hospital** | **Local Currency** | **Mean Cost of Care - No-PPH** | **Mean Cost of Care - PPH** | **Increased cost of care for PPH over No-PPH** | **% Increase in Cost of Care** |
| --- | --- | --- | --- | --- | --- | --- |
| India | Bagalkot | INR | 489·71 | 843·62 | 353·91 | 72% |
| India | Belgaum | INR | 489·71 | 1,657·06 | 1,167·35 | 238% |
| India | Bijapur | INR | 739·37 | 1,120·71 | 381·34 | 52% |
| India | Cuttack | INR | 859·40 | 1,181·76 | 322·36 | 38% |
| India | Nagpur | INR | 897·12 | 1,392·32 | 495·20 | 55% |
| Kenya | Nairobi | KES | 2,403·46 | 6,864·84 | 4,461·38 | 186% |
| Nigeria | Akure | NGN | 992·78 | 4,729·24 | 3,736·46 | 376% |
| Nigeria | Ibadan | NGN | 7,198·56 | 19,425·99 | 12,227·44 | 170% |
| Uganda | Kampala | UGX | 53,423·08 | 69,807·69 | 16,384·62 | 31% |

Table S1c - Surgical interventions

| **Country** | **Hospital Name** | **Exchange Rate** | **Intervention Price LC** | **Drug Tray Price LC** | **Intervention Price USD** | **Drug Tray Price USD** |
| --- | --- | --- | --- | --- | --- | --- |
| **Suturing cervix/ high vaginal tear** | | | | | | |
| India | Bagalkot | 0.01458 | 2,425 | 1,162 | 35.4 | 16.9 |
| India | Belgaum | 0.01458 | 4,054 | 720 | 59.1 | 10.5 |
| India | Bijapur | 0.01458 | 3,520 | 999 | 51.3 | 14.6 |
| India | Cuttack | 0.01458 | 1,779 | 579 | 25.9 | 8.4 |
| India | Nagpur | 0.01458 | 1,898 | 428 | 27.7 | 6.2 |
| Kenya | Nairobi | 0.00984 | 27,280 | 2,500 | 268.4 | 24.6 |
| Nigeria | Akure | 0.00277 | 6,902 | 2,900 | 19.1 | 8 |
| Nigeria | Ibadan | 0.00277 | 27,428 | 13,329 | 76 | 36.9 |
| Uganda | Kampala | 0.00026 | 96,562 | 55,760 | 25.1 | 14.5 |
| **Intrauterine balloon/ condom tamponade** | | | | | | |
| India | Cuttack | 0.01458 | 954 | 362 | 13.9 | 5.3 |
| Kenya | Nairobi | 0.00984 | 27,340 | 2,790 | 269 | 27.5 |
| Nigeria | Akure | 0.00277 | 6,252 | 2,500 | 17.3 | 6.9 |
| Uganda | Kampala | 0.00026 | 49,673 | 46,567 | 12.9 | 12.1 |
| **Exploration of uterine cavity** | | | | | | |
| India | Belgaum | 0.01458 | 2,980 | 944 | 43.4 | 13.8 |
| India | Bijapur | 0.01458 | 2,859 | 999 | 41.7 | 14.6 |
| Nigeria | Akure | 0.00277 | 4,738 | 3,100 | 13.1 | 8.6 |
| Nigeria | Ibadan | 0.00277 | 17,309 | 10,250 | 47.9 | 28.4 |
| **Uterine or hypogastric ligation** | | | | | | |
| Nigeria | Akure | 0.00277 | 25,846 | - | 71.6 | 0.0* |
| **Hysterectomy** |  |  |  |  |  |  |
| India | Bijapur | 0.01458 | 7,757 | 2,867 | 113.1 | 41.8 |
| Kenya | Nairobi | 0.00984 | 59,053 | 7,645 | 581.1 | 75.2 |
| Nigeria | Ibadan | 0.00277 | 80,246 | 42,659 | 222.3 | 118.2 |
| Uganda | Kampala | 0.00026 | 214,247 | 80,876 | 55.7 | 21 |
| **Manual removal of placenta** | | | | | | |
| India | Bagalkot | 0.01458 | 1,857 | 2,014 | 27.1 | 29.4 |
| India | Bijapur | 0.01458 | 2,859 | 999 | 41.7 | 14.6 |
| India | Cuttack | 0.01458 | 1,123 | 738 | 16.4 | 10.8 |
| India | Nagpur | 0.01458 | 625 | 1,442 | 9.1 | 21 |
| Kenya | Nairobi | 0.00984 | 19,498 | 4,930 | 191.9 | 48.5 |
| Nigeria | Akure | 0.00277 | 3,353 | 2,900 | 9.3 | 8 |
| Nigeria | Ibadan | 0.00277 | 30,398 | - | 84.2 | 0.0* |
| Uganda | Kampala | 0.00026 | 54,073 | 60,981 | 14.1 | 15.9 |
| **Bimanual compression** | | | | | | |
| India | Bagalkot | 0.01458 | 433 |  | 6.3 | 0 |
| India | Belgaum | 0.01458 | 1,480 |  | 21.6 | 0 |
| India | Bijapur | 0.01458 | 917 |  | 13.4 | 0 |
| India | Cuttack | 0.01458 | 759 |  | 11.1 | 0 |
| India | Nagpur | 0.01458 | 735 |  | 10.7 | 0 |
| Kenya | Nairobi | 0.00984 | 758 |  | 7.5 | 0 |
| Nigeria | Akure | 0.00277 | 1,125 |  | 3.1 | 0 |
| Nigeria | Ibadan | 0.00277 | 529 |  | 1.5 | 0 |
| Uganda | Kampala | 0.00026 | 14,952 |  | 3.9 | 0 |

* All drugs and consumables are included in a surgical pack

Table S1d - Blood transfusions

| **Country** | **Hospital name** | **Exchange Rate** | **Consumables, investigations, fees (LCY)**  **(A)** | **Blood fees + labour cost (LCY)**  **(B)** | **Total first unit price (LCY) (A+B)** | **Total first unit price (USD)** |
| --- | --- | --- | --- | --- | --- | --- |
| **Blood** | | | | | | |
| India | Bagalkot | 0.01458 | 370 | 1,596 | 1,966 | 28.67 |
| India | Belgaum | 0.01458 | 214 | 930 | 1,144 | 16.68 |
| India | Bijapur | 0.01458 | 249 | 1,562 | 1,810 | 26.39 |
| India | Cuttack | 0.01458 | 70 | 699 | 769 | 11.21 |
| India | Nagpur | 0.01458 | 295 | 657 | 952 | 13.88 |
| Kenya | Nairobi | 0.00984 | 130 | 4,091 | 4,221 | 41.53 |
| Nigeria | Akure | 0.00277 | 200 | 3,425 | 3,625 | 10.04 |
| Nigeria | Ibadan | 0.00277 | 1,197 | 8,300 | 9,497 | 26.31 |
| Uganda | Kampala | 0.00026 | 11,511 | 11,973 | 23,484 | 6.11 |
| **Packed red cells** | | | | | | |
| India | Bagalkot | 0.01458 | 370 | 1,246 | 1,616 | 23.56 |
| India | Belgaum | 0.01458 | 214 | 920 | 1,134 | 16.54 |
| Uganda | Kampala | 0.00026 | 11,511 | 10,413 | 21,924 | 5.7 |
| **Fresh frozen plasma** | | | | | | |
| India | Bagalkot | 0.01458 | 370 | 946 | 1,316 | 19.19 |
| Kenya | Nairobi | 0.00984 | 130 | 3,591 | 3,721 | 36.61 |
| Nigeria | Ibadan | 0.00277 | 1,197 | 4,700 | 5,897 | 16.33 |

Table S1e - Additional Uterotonics

| **Country** | **Hospital Name** | **Exchange Rate** | **Price LCY** | **Price USD** | **Unit** |
| --- | --- | --- | --- | --- | --- |
| **Oxytocin** | | | | | |
| India | Bagalkot | 0.01458 | 18 | 0.26 | 5 IU |
| India | Belgaum | 0.01458 | 18 | 0.26 | 5 IU |
| India | Bijapur | 0.01458 | 18 | 0.26 | 5 IU |
| India | Cuttack | 0.01458 | 18 | 0.26 | 5 IU |
| India | Nagpur | 0.01458 | 18 | 0.26 | 5 IU |
| Kenya | Nairobi | 0.00984 | 40 | 0.39 | 5 IU |
| Nigeria | Akure | 0.00277 | 200 | 0.55 | 10 IU |
| Nigeria | Ibadan | 0.00277 | 500 | 1.39 | 10 IU |
| Uganda | Kampala | 0.00026 | 205 | 0.05 | 10 IU |
| **Ergometrine** | | | | | |
| India | Bagalkot | 0.01458 | 13 | 0.19 | 200 ug |
| India | Belgaum | 0.01458 | 15 | 0.22 | 200 ug |
| India | Bijapur | 0.01458 | 13 | 0.19 | 200 ug |
| India | Cuttack | 0.01458 | 14 | 0.2 | 200 ug |
| India | Nagpur | 0.01458 | 15 | 0.22 | 200 ug |
| Kenya | Nairobi | 0.00984 | 200 | 1.97 | 500 ug |
| Nigeria | Akure | 0.00277 | 150 | 0.42 | 500 ug |
| Nigeria | Ibadan | 0.00277 | 400 | 1.11 | 500 ug |
| Uganda | Kampala | 0.00026 | 1,286 | 0.33 | 500 ug |
| **Misoprostol** | | | | | |
| India | Bagalkot | 0.01458 | 18 | 0.26 | 200 mcg |
| India | Belgaum | 0.01458 | 18 | 0.26 | 200 mcg |
| India | Bijapur | 0.01458 | 18 | 0.26 | 200 mcg |
| India | Cuttack | 0.01458 | 17 | 0.25 | 200 mcg |
| India | Nagpur | 0.01458 | 17 | 0.25 | 200 mcg |
| Kenya | Nairobi | 0.00984 | 20 | 0.2 | 200 mcg |
| Nigeria | Akure | 0.00277 | 200 | 0.55 | 200 mcg |
| Nigeria | Ibadan | 0.00277 | 65 | 0.18 | 200 mcg |
| Uganda | Kampala | 0.00026 | 256 | 0.07 | 200 mcg |
| **Carboprost (PGF2α) (Prostodin)** | | | | | |
| India | Bagalkot | 0.01458 | 175 | 2.55 | 250 mcg |
| India | Belgaum | 0.01458 | 106 | 1.55 | 250 mcg |
| India | Bijapur | 0.01458 | 106 | 1.55 | 250 mcg |
| India | Cuttack | 0.01458 | 147 | 2.14 | 250 mcg |
| India | Nagpur | 0.01458 | 106 | 1.55 | 250 mcg |

Table S1f - Cost of stay - 1 Day in Labour Ward

| **Country** | **Hospital Name** | **Exchange rate** | **Price LCY** | **Price USD** |
| --- | --- | --- | --- | --- |
| India | Bagalkot | 0.01458 | 80 | 1.17 |
| India | Belgaum | 0.01458 | 100 | 1.46 |
| India | Bijapur | 0.01458 | 150 | 2.19 |
| India | Cuttack | 0.01458 | 280 | 4.08 |
| India | Nagpur | 0.01458 | 280 | 4.08 |
| Kenya | Nairobi | 0.00984 | 1,200 | 11.81 |
| Nigeria | Akure | 0.00277 | 500 | 1.39 |
| Nigeria | Ibadan | 0.00277 | 1,950 | 5.4 |
| Uganda | Kampala | 0.00026 | 30,012 | 7.8 |

Table S1g - Concomitant Drug calculations

| **Column name** | **Description** | **Excel Formula** |
| --- | --- | --- |
| Medname | Name of drug administered | n/a - From trial data |
| Conc, unit, UNIT2 | Quantity administered (amount and unit) | n/a - From trial data |
| Frequency | Administration frequency | n/a - From trial data |
| Route | Drug administration route | n/a - From trial data |
| DOD Baby | Date of delivery | n/a - From trial data, imported from Delivery worksheet |
| Datestart | Start of treatment | n/a - From trial data |
| Treatment Start | Drug treatments were only costed as of the date of delivery (DOD). If the “datestart” was on the DOD or later, this is what was used. If it was prior, then the DOD was used. | =IF(datestart>=DOD baby, datestart, DOD baby) |
| Dateend | End of drug treatment, if treatment ended prior to hospital discharge. If treatment was ongoing after discharge, this was left blank and “1” was marked in the “Ongoing” column. | n/a - From trial data |
| Date discharge | Patient discharge date. | n/a - From trial data, imported from delivery worksheet |
| Ongoing | Indicates if treatment is ongoing after discharge. | n/a - From trial data |
| Treatment end | If a treatment is ongoing, the discharge date is used as the end of treatment date. | =IF(ongoing=1,date discharge,dateend) |
| Price/ single unit | Price of each tablet, ampoule, bottle etc.… | From price lists/ site committee |
| Single unit concentration | Drug single unit (ex. 500mg tablet) | From site committee |
| # units/ dose | Number of single units in the administered dose (ex. 1g/0.5g=2 tablets)  Administered concentration/ single unit concentration | =IF(ISERROR([@concentration]/[@[Single unit concentration]]),"",[@concentration]/[@[Single unit concentration]]) |
| Dose price | Price of administered dose | =IF(ISERROR([@[Price/ single unit]]*[@['# units/ dose]]),"",[@[Price/ single unit]]*[@['# units/ dose]]) |
| Doses/ day | Number of times/ day the drug was administered during the treatment period | =IF(ISERROR([@[Price/ single unit]]*[@['# units/ dose]]),"",[@[Price/ single unit]]*[@['# units/ dose]]) |
| Daily price | Treatment price per day | =IF(ISERROR([@[Dose price]]*[@[Doses/ day]]),"",[@[Dose price]]*[@[Doses/ day]]) |
| Total treatment # days | Length of treatment (days) of all treatments | =DATEDIF([@datestart],[@[Treatment end]],"d")+1 |
| Treatment days from birth (if treatment started earlier) | Length of treatment (days) from date of delivery, if treatment started before | =IF([@[Treatment end]]-[@[Treatment Start]]<0,0,[@[Treatment end]]-[@[Treatment Start]]+1) |
| Total treatment cost local currency | Cost of treatment in local currency | =IF(ISERROR([@[Daily price]]*[@[Treatment days from birth (if treatment started earlier)]]),0,[@[Daily price]]*[@[Treatment days from birth (if treatment started earlier)]]) |
| Not for PPH | Indicates if treatment ended before the DOD, in which case it was not included in costing | =IF(Treatment end<DOD Baby, "NOT for PPH","") |
| Include/ Exclude | All treatments which began before the DOD were excluded. | =IF([@datestart]<[@[DOD Baby]],"Exclude","Include") |
| UniqueIdentifier |  | =CONCATENATE([@[WHO Drug Name]],"-",[@unit],"-",[@[Single unit concentration]],"-",[@[Single concentration unit]],"-",[@hospitalcode],"-",[@Centre]) |
| UnitPrice_USD |  | =VLOOKUP([@Centre],tblCountryData,5,FALSE)*[@[Price/ single unit]] |
| Total_Treatment_Cost_USD |  | =VLOOKUP([@Centre],tblCountryData,5,FALSE)*[@[Total_treatment_cost_local_currency]] |
